# “*Lachak*” and the Normalization of Nonbelonging: Skilled Migration, Symbolic Capital, and Settlement in Canada

**DOI:** 10.1111/cars.70049

**Published:** 2026-08-04

**Authors:** Hammad Khan

**Affiliations:** ^1^ Department of Sociology University of Toronto Toronto Canada

**Keywords:** belonging, deskilling, immigration, nonbelonging, symbolic capital

## Abstract

This article examines how skilled Pakistani newcomers to Canada experience and make sense of the loss of recognition associated with their prior professional identities following migration. Drawing on 43 life‐history interviews in the Greater Toronto Area, I use the Urdu word *lachak* (flexibility) to describe how my interlocutors lower expectations, accept downward mobility, and reorient their identities in order to move forward with settlement. I argue that *lachak* captures how symbolic capital loss becomes embedded in everyday self‐understandings, producing an embodied and often obscured sense of nonbelonging. The analysis also demonstrates that these processes are structured through gendered differences across professional and domestic domains. By foregrounding *lachak*, the article shows how nonbelonging persists through the normalization of diminished recognition in settlement.

## RÉSUMÉ

Cet article examine comment les nouveaux arrivants pakistanais qualifiés au Canada vivent et interprètent la perte de reconnaissance liée à leurs identités professionnelles antérieures à la suite de leur migration. En m'appuyant sur 43 entretiens biographiques menés dans la région du Grand Toronto, j'utilise le mot ourdou *lachak* (flexibilité) pour décrire comment mes interlocuteurs reviennent sur leurs attentes, acceptent une mobilité descendante et réorientent leurs identités afin de poursuivre leur processus d’établissement. Je soutiens que le *lachak* rend compte de la manière dont la perte de capital symbolique s'ancre dans la perception que l'on a de soi au quotidien, engendrant un sentiment incarné et souvent dissimulé de non‐appartenance. L'analyse démontre également que ces processus sont structurés par des différences liées au genre, tant dans la sphère professionnelle que domestique. En mettant en avant le *lachak*, cet article montre comment le sentiment de non‐appartenance persiste à travers la normalisation d'une reconnaissance réduite au cours de l'installation.

## 1 Introduction

“I don't want to seem ungrateful, but you're telling people there that they have the points, and here, no one sees you as an engineer. Here, you're an Uber driver. Then you too become an Uber driver. That becomes your identity. An engineer in Pakistan, and an Uber driver in Canada.”

Before migrating to Canada from Karachi, Anwar worked as a civil engineer for the municipal government and held a degree from one of Pakistan's most prestigious engineering schools. In Pakistan, this professional identity carried status, recognition, and a clear sense of self and social position. After arriving in Canada, that recognition disappeared abruptly. Reflecting on this shift, Anwar explained that he no longer “recognized” himself, requiring him to adjust to that change during settlement.

Despite being selected for entry through a points‐based ranking system that privileges immigrants’ symbolic capital, many immigrants find that these forms of capital carry limited value in Canada (Creese and Wiebe 2012; Karki 2025). While deskilling for immigrants is well documented (Banerjee et al. 2024; Guo 2015; Ng and Gagnon 2020), less attention has been paid to how newcomers interpret this loss, and how it becomes normalized in settlement. To address this gap, this article asks two questions: how do Pakistani newcomer immigrants navigate the loss of recognition they encounter after arrival, and what do these accounts reveal about how adjustment becomes a mechanism through which nonbelonging is produced and sustained within the settlement process, including along gendered lines?

It is within this context that the term *lachak* 1 emerges through my interlocutors’ accounts as they make sense of misrecognition in settlement. *Lachak* refers to a moral and practical orientation through which racialized skilled newcomers lower expectations, accept downward mobility as a normal part of settlement, and reorient their sense of identity and belonging during their settlement. The use of Urdu in naming *lachak* is analytically significant as it foregrounds how participants interpret and articulate these processes themselves and on their own terms. Attending to this language shows how flexibility becomes necessary for navigating misrecognition, tying belonging to the willingness to adjust.

Drawing on Yuval‐Davis’ (2006, 2011) concept of the politics of belonging and Korteweg and Yurdakul's (2024) formulation of nonbelonging as an actively produced condition, I conceptualize nonbelonging as a patterned and embodied feature of settlement rather than simply the absence of belonging. I bring this into conversation with Bourdieu's (1984, 1991) theory of symbolic capital and symbolic violence to show how misrecognition is not only imposed structurally but internalized through everyday practices of adjustment. Bringing these frameworks together, I show that nonbelonging emerges as skilled Pakistani newcomers come to interpret misrecognition as something to be managed through adjustment.

This article makes three contributions to scholarship on migration and belonging. First, it introduces *lachak* as an empirically grounded analytic that captures how adjustment is not simply a response to structural constraints but a socially learned orientation through which diminished recognition is normalized. Second, it extends existing work on deskilling and nonbelonging by showing how these conditions persist not only through outright exclusion but through the very practices that enable settlement, rendering them less visible. Third, by foregrounding gendered differences, it demonstrates how these processes unfold unevenly across both professional and domestic domains.

This study draws on 43 in‐depth, life‐history interviews with recent Pakistani immigrants in the Greater Toronto Area (GTA) to examine how participants narrate disruptions to their professional identities and interpret the adjustments that follow. While participants shared a national‐origin background, they differed across gender, class background, language use, and migration trajectories, which shaped how recognition and belonging were experienced in settlement. The sections that follow outline the theoretical framework, situate the study within scholarship on immigrant settlement, and then turn to the empirical analysis.

## 2 Theoretical Framework

Drawing on Nira Yuval‐Davis (2006), I conceptualize belonging as structured through boundary‐making and recognition. This framework has been further developed through attention to power and intersectionality (Yuval‐Davis 2016), shifting attention away from formal inclusion toward the everyday practices through which membership is organized and experienced. This perspective also highlights how these same processes can produce forms of exclusion and conditional inclusion, creating the conditions through which nonbelonging emerges. Building on this, Korteweg and Yurdakul (2024) conceptualize nonbelonging not simply as the absence of belonging, but as an actively produced and embodied condition. As they note, nonbelonging enters “into circulation through human interaction at the everyday level” (Korteweg and Yurdakul 2024, 296–297; see also Özvatan et al. 2023). This framing highlights how individuals may experience forms of inclusion while simultaneously encountering embodied misrecognition.

I extend and combine these frameworks by examining how such embodied forms of nonbelonging may also become difficult to recognize, not only because they are lived through everyday practices of adjustment, but because they are sustained through the very practices that enable settlement. This shifts attention from nonbelonging as a visible condition of exclusion to one that may be obscured by processes of adjustment through the politics of belonging.

To explain how these processes are internalized and sustained, I bring these frameworks into conversation with Bourdieu's theory of symbolic capital and symbolic violence. Bourdieu (1984, 1986) defines symbolic capital as forms of recognition and legitimacy that depend on their valuation within a given social field, referring to them as “another name for distinction” (Bourdieu 1985, 731). For immigrants, credentials and experience that carry status in one context may be misrecognized or devalued in another, reshaping their social positioning based on perceived legitimacy. When these hierarchies are internalized as natural or inevitable, they take the form of symbolic violence, through which unequal positioning comes to be interpreted as normal or justified (Bourdieu 1984; Thapar‐Björkert et al. 2016).

Bringing these frameworks together, I conceptualize nonbelonging as the lived effect of symbolic violence, where the loss of symbolic capital is internalized through everyday practices of adjustment. Yuval‐Davis’ (2011) politics of belonging locates how inclusion is structured through recognition and boundary‐making, while Korteweg and Yurdakul's (2024) formulation of nonbelonging foregrounds how exclusion is actively produced and embodied. Bourdieu's (1984, 1991) framework explains how these conditions are internalized, showing how misrecognition becomes incorporated into everyday practices and self‐understandings. Within this synthesis, misrecognition refers to the devaluation of participants’ credentials, experience, and prior social worth, while nonbelonging names what emerges when that devaluation is absorbed into participants’ sense of self and perceived place in Canada. It emerges among newcomers who are incorporated into Canadian settlement through immigration status, work, and family life, yet experience this incorporation as conditional because the recognition through which they previously understood themselves no longer carries the same value.

In this framing, nonbelonging persists alongside a contingent form of inclusion, wherein flexibility in the face of misrecognition becomes the price of settlement, sustaining the very conditions that require it. Unlike exclusion or marginalization, which emphasize external positioning, nonbelonging captures how these conditions are lived, internalized, and reproduced through everyday practices of adjustment. *Lachak* is the practice through which participants negotiate belonging under conditions of misrecognition, allowing them to move forward in settlement while also normalizing the diminished recognition through which nonbelonging is produced.

## 3 Literature Review

### 3.1 The Points System and the Institutional Mismatch of Symbolic Capital

Newcomers’ settlement experiences, and their perceptions of their position in Canada, are shaped by broader institutional processes that unevenly recognize and value forms of symbolic capital. The points‐based immigration system provides a key entry point into these dynamics. It is often framed as a race‐neutral and meritocratic model, emerging in the context of broader shifts away from explicitly exclusionary immigration policies (Bloemraad 2015; FitzGerald and Cook‐Martín 2014). It evaluates prospective immigrants based on education, work experience, language proficiency, and age. While framed as objective, this system continues to operate within white‐settler logics that shape what forms of capital are recognized as legitimately “Canadian” (Tannock 2011; Thomas 2023).

These dynamics extend beyond selection into settlement itself, where institutional practices systematically undervalue immigrants’ credentials and experience. Even today, with the Express Entry program for skilled workers and the Comprehensive Ranking System, “Canadian experience” continues to be privileged over foreign credentials (Banerjee, Zhang, and Amarshi 2025; Bhuyan et al. 2017; Zhang et al. 2023), contributing to the persistent devaluation of immigrants’ qualifications (Grant and Nadin 2007; Imai et al. 2019; Konnikov 2023). Even when immigrants pursue additional Canadian education to compensate for this disadvantage, many continue to experience downward occupational mobility (Adamuti‐Trache et al. 2013). Taken together, although this literature highlights how institutional processes structure the devaluation of immigrants’ symbolic capital, it offers limited insight into how these dynamics are interpreted and navigated in settlement.

### 3.2 From Deskilling to Embodied Nonbelonging

This institutional mismatch produces patterns of deskilling among racialized immigrants in Canada (Bauder 2003; Ferrer and Riddell 2008; Guo 2015; Konnikov 2023). Highly educated newcomers often find that the education, experience, and status for which they were selected carry limited value in Canada (Somerville and Walsworth 2010). Many are pushed into precarious work or required to retrain for entry‐level positions, producing persistent downward mobility across multiple immigrant groups (Cornelissen and Turcotte 2020; Crea‐Arsenio et al. 2022; Lamb et al. 2021; Picot et al. 2016). What seems like a problem of credential recognition is, in practice, a broader reordering of social worth, in which symbolic capital accumulated in the country of origin loses legitimacy in Canada.

These experiences are more than economic setbacks; research shows that these experiences generate frustration, stress, and disorientation, especially among immigrants who migrate with expectations of professional continuity (Simich et al. 2005; Simich et al. 2006; Smith and Ley 2008; Subedi and Rosenberg 2016). Labor market experiences can also reshape broader perceptions of Canada, as everyday realities unsettle pre‐migration imaginaries of the country (Hande et al. 2020). As Kazemipur and Nakhaie (2014) argue, attachment to Canada is closely tied to experiences of recognition in settlement, suggesting that labor market positioning is deeply intertwined with questions of belonging.

Despite this established work, settlement continues to be framed through the logic of integration, where early difficulties are treated as temporary barriers to be overcome with time through retraining or credential acquisition (Chuatico et al. 2023; Walters et al. 2007). Sense of belonging measures are often mobilized as evidence of successful integration (Statistics Canada 2016; 2023), reinforcing the assumption that settlement challenges are temporary. While recent scholarship critiques this logic (Korteweg 2017; Korteweg and Yurdakul 2024; Schinkel 2018), further empirical work is needed to examine how misrecognition is normalized as newcomers adjust around these barriers.

### 3.3 Gendered Dimensions and the Case of Skilled Pakistani Newcomers

Existing research shows that processes of deskilling and labor market exclusion are shaped by gender, with immigrant women often experiencing particularly acute forms of marginalization (Amoako et al. 2024). These studies highlight how barriers to credential recognition intersect with gender, race, and immigration status, contributing to persistent inequalities in employment outcomes. However, much of this work pays less attention to how these dynamics extend beyond professional contexts into the reorganization of social roles and identities. Research on Pakistani immigrant women in Canada shows that these barriers are often accompanied by shifts into unpaid domestic roles and reconfigured household dynamics (Zaman 2010). This shift is particularly significant when considered in relation to gendered expectations in Pakistan, where women's participation in paid work is frequently tied to forms of autonomy, including increased bargaining power and decision‐making within the household (Tarar et al. 2022). Together, these dynamics suggest that barriers to employment can reshape gendered and classed forms of recognition beyond the labor market, particularly where migration reorganizes paid work, household labor, and access to domestic support.

Such processes are especially visible in the experiences of skilled Pakistani newcomers to Canada, many of whom arrive through economic immigration pathways designed to select for education, language ability, and prior professional experience, producing strong expectations of recognition upon arrival (Karki 2025). Despite this, they encounter well‐documented patterns of credential devaluation and labor market deskilling (Banerjee et al. 2024; Bauder 2003; Lu and Hou 2020). For Pakistani immigrants specifically, research has documented experiences of underemployment, frustration, and mental health strain tied to these barriers (Akram‐Pall and Moodley 2016; Boyd and Tian 2018; Jibeen and Khalid 2010).

These experiences are also shaped by intersecting social differences, including gender, class, and professional background (Khan and Watson 2005; Nichols et al. 2018; Sharma 2006). While Pakistani newcomers are not analytically exceptional, they provide a strategically bounded case for examining how expectations of recognition, often shaped prior to arrival through transnational information flows (Shuva 2024), collide with their systematic devaluation in Canada. This makes them a particularly useful group for examining how processes of adjustment unfold across both professional and everyday contexts, and how these processes are structured in gendered terms.

Overall, such patterns point to the need to move beyond framing settlement outcomes as temporary barriers, toward examining how they are incorporated into everyday understandings of belonging. While existing research has documented these structural dynamics, less attention has been paid to how skilled immigrants come to interpret and accommodate them as a normal and taken‐for‐granted part of settlement. The present study addresses this gap by examining how adjustment itself becomes a key mechanism through which these conditions are lived and sustained.

## 4 Methods

I draw on 43 life‐history interviews with Pakistani immigrants in the GTA who arrived through points‐based economic pathways. Participants were adults (ages 25–48) who had been in Canada for five years or fewer. The sample included 25 men and 18 women from middle‐ to upper‐class skilled professional backgrounds, most originating from major urban centers like Karachi, Lahore, and Islamabad. Focusing on Pakistani immigrants is an analytical choice intended to illuminate broader processes rather than to treat this group as exceptional. As a group that is highly represented within Canada's skilled immigration streams (Statistics Canada 2024) and often arrives with strong expectations of professional continuity, their experiences reflect the tension between formal selection criteria and labor market outcomes. This makes them a particularly revealing case for examining how symbolic capital loss is interpreted in contexts where migrants are formally positioned for successful integration.

I recruited participants through purposive and snowball sampling, beginning with my community networks in the GTA, then expanding the sample through participant referrals. Participants met three selection criteria: 1) they migrated from Pakistan with the intention of permanently settling in Canada, 2) they entered through points‐based immigration pathways, and 3) they had been in Canada for five years or fewer. Although participants shared a national‐origin background, the sample reflects variation across gender, class trajectories, linguistic repertoires, and professional fields.

I used life‐history interviews to examine migration as a temporally continuous and socially embedded process (Adriansen 2012; Cole and Knowles 2001). Interviews followed a semi‐structured guide focused on migration trajectories, labor market experiences, credential recognition, and perceptions of belonging. Rather than asking directly about “nonbelonging,” I invited participants to narrate their experiences in their own terms, allowing themes of recognition, adjustment, and identity to emerge through their accounts. This enabled me to analyze how participants connected past recognition in Pakistan to their settlement experiences in Canada. This approach aligns with research that treats migration as occurring across interconnected social fields (Bourdieu 1990), better suited to understanding the layered and intersectional dimensions of settlement (Brotman et al. 2020; Ghorashi 2008). Interviews were conducted primarily in English, Urdu, and Punjabi, with occasional use of colloquial Hindi and Pashto depending on participants’ preferences. I conducted, transcribed, and translated all interviews myself. When participants used culturally specific expressions, I retained their phrasing and translated it as closely as possible to preserve contextual meaning.

I analyzed the interviews using an inductive, iterative coding process. I began with broad, open coding focused on participants’ migration narratives, including expectations prior to arrival, early settlement experiences, and general accounts of belonging and adjustment. Through this initial coding, more specific themes emerged around labor market experiences, credential recognition, and participants’ shifting understandings of value, worth, and status in Canada. I compared these themes across interviews to identify recurring patterns in how participants described discrepancies between their professional lives in Pakistan and their experiences in Canada.

As I refined the analysis, I noted the repeated emphasis on flexibility across interviews. I then returned to the data to examine how participants invoked this in relation to patterns of misrecognition and adjustment. Rather than treating *lachak* as an isolated expression, I analyzed how it functioned as a shared interpretive framework through which participants made sense of their experiences, allowing me to trace how symbolic capital loss was incorporated into their understandings of settlement itself. Although this study draws on 43 interviews, I present excerpts from a smaller number of analytically generative cases that illustrate recurring patterns across the interviews, allowing for closer analysis of how participants normalize adjustment as part of settlement.

Finally, my position as a first‐generation Pakistani immigrant shaped both access to participants and the dynamics of the interviews. My own migration experiences informed part of the motivation for this study and created an affective connection with participants, which often facilitated rapport and openness. This was evident in moments where participants drew on assumed shared understanding, using phrases such as “*aap ko toh pata hai*” or “*aap toh jaantay hain*,”^2^ or shifting to the collective pronoun “*hum* (we),” even when describing their own experiences. Participants also frequently used the term *lachak* without explanation, expecting that I would understand its meaning and implications. While this familiarity enabled depth, it also required careful reflexive attention to moments where shared assumptions might obscure differences in experience. To address this, I treated instances of assumed understanding as analytically significant, prompting further clarification during interviews where necessary and revisiting these moments during analysis to distinguish between taken‐for‐granted meanings and broader patterns.

This research received ethical approval from the author's university Research Ethics Board.

## 5 Findings

The following sections examine how highly skilled Pakistani newcomers experienced the loss of symbolic capital following their arrival, and how this reshaped their sense of identity, belonging, and nonbelonging. The analysis traces how this loss is interpreted and incorporated into understandings of settlement itself, illustrating how symbolic violence operates through everyday practices of settlement, as misrecognition is internalized. Section 5.1 examines how symbolic capital loss reshapes professional identity and produces a sense of conditional belonging, through which experiences of nonbelonging begin to emerge. Section 5.2 shows how participants make sense of these disruptions through the language of *lachak* (flexibility), and Section 5.3 demonstrates how these processes are structured through gendered differences. Taken together, the analysis shows how diminished recognition comes to be accommodated as part of settlement, allowing nonbelonging to persist in less visible ways.

### 5.1 Lost Recognition: Symbolic Capital, Professional Identity, and Belonging

As prior research has established, the non‐recognition of foreign credentials often produces downward occupational mobility among highly educated immigrants in Canada (Bauder 2003; Ferrer and Riddell 2008; Somerville and Walsworth 2010). This section examines how the loss of symbolic capital across national contexts reshapes participants’ sense of professional identity and structures their national belonging in Canada. Across interviews, participants compared the recognition attached to their credentials in Pakistan with its diminished value in Canada. These comparisons show that symbolic capital loss is experienced not only as labor market disadvantage, but as a disruption of self, status, and social position.

Nargis’ and Anwar's experiences were especially illustrative of these dynamics. In Karachi, Nargis worked as a nurse, and Anwar was employed as a civil engineer for the municipal government. Both held degrees from two of Pakistan's most prestigious institutions: the University of Karachi and Nadirshaw Edulji Dinshaw University of Engineering and Technology (NED University), respectively. Yet the prestige associated with their alma maters and professions carried little weight in Canada. When reflecting on this loss, Anwar confessed that he did not “recognize” himself in Canada. His decision to switch into English to make this point underscored the emphasis he placed on the dissonance he now felt when reflecting on his sense of belonging and identity in Canada:

I don't want to seem ungrateful, but you're telling people there that they have the points, and here, no one sees you as an engineer. Here, you're an Uber driver. Then you too become an Uber driver. That becomes your identity. An engineer in Pakistan, and an Uber driver in Canada. We are Canadian, but not a Canadian engineer, or a Canadian nurse. A Canadian Uber driver, and a Canadian *store worker*. That's what you become. Then you tell us, how can you feel happy in these circumstances, or that *aap yahan ke ho?* (Literally translates to being of or from a place with connotations of belonging to that place)

Introduced earlier, Anwar's account directly links national belonging with occupational prestige and professional recognition. His professional identity as a civil engineer was a symbol of status and legitimacy that shaped how he understood himself in Pakistan. In Canada, that recognition was effectively erased, and his work as an Uber driver signifies his place here now. The tension between being “Canadian” while not being recognized as a Canadian professional reveals how belonging can remain conditional even when assumed inclusion through immigration and citizenship is formally present. For Anwar, the loss of symbolic capital disrupts this recognition, producing a fractured sense of identity across national contexts. Nargis echoed this sense of rupture, but highlighted the bureaucratic dimensions through which these barriers are navigated:

Maybe it's possible for me [to get accreditation], but maybe it's not for him. That part he has to leave behind.

Here, Nargis recognized that structural pathways to professional re‐entry were unevenly distributed across sectors. While she believed that nursing might eventually allow her to regain some professional recognition through recredentialing, she acknowledged that Anwar's engineering credentials were far less likely to be recognized in Canada. Their reflections illustrate how symbolic capital loss is accommodated as part of settlement itself.

Despite being warned by family members in Canada, Anwar recalled being “optimistic” that he would work as a civil engineer. While he later described himself as financially stable, he emphasized that this relative stability had not erased his dissonance or helped him regain his lost sense of self:

Over there, to be a civil engineer for the government, that's an identity, and the truth is, I *am* those things (Italicized for Anwar's emphasis). I will always be that because I achieved that. But people will not see me as that here. Then you don't feel fully like you're from here.

Anwar's comment here highlights a central contradiction in the settlement trajectories of highly skilled immigrants. His professional identity remains meaningful to him personally, yet it is no longer recognized by the institutions and labor markets that structure belonging in Canada. This disjunction illustrates how symbolic capital loss extends beyond employment outcomes to shape the way immigrants experience their place within the national community.

Other interlocutors similarly described the nature of symbolic capital loss in relational terms. Hussain, for instance, drew on his international mobility and high‐prestige career history to underscore the depth of this shift. Having worked at Coca‐Cola, Suzuki, and Red Bull's corporate office in Dubai, which he described as “the best company in the world for energy drinks”, Hussain arrived in Canada confident that his credentials would carry weight. Yet these markers of professional distinction were not recognized in Canada:

[…] The problem in Canada is the transferability of skills. They don't transfer your skills easily. And you're not able to work in the field in which you're qualified, through which you come here. I've seen an Indian Ph.D. working in a store here, I've seen an Indian dentist working in a warehouse here, I've seen an Indian engineer working in a warehouse here. I'm a Pakistani accountant; I'm working at a warehouse right now. […]

Hussain's narrative shows this relational element further. Observing other highly educated immigrants from across South Asia in similar positions reinforced his understanding that downward mobility was not an isolated experience but part of a broader pattern affecting racialized immigrants like him. Nonetheless, these structural dynamics were experienced most acutely through the contrast between recognition attached to his career abroad and the diminished status of his work in Canada, revealing how structural patterns are interpreted as individual responsibility. In this sense, symbolic violence operates by reframing structural misrecognition as an individual problem, obscuring the broader conditions that produce it.

Zahid, who held a master's degree in biotechnology and previously earned high incomes, likewise abandoned his professional trajectory in Canada and retrained as a nurse. He described this shift as motivated by a desire to represent Pakistan, which he referred to as “my country,” in a positive way. Zahid's framing reveals how symbolic capital loss can become intertwined with questions of national representation. Rather than interpreting downward mobility solely as structural exclusion, he also understood his adjustment as a form of responsibility toward both his family and his country of origin, further illustrating how adjustment is reframed as a personal responsibility. Symbolic capital loss is reinterpreted as an obligation, transforming what might be understood as structural exclusion into an individual imperative to adjust.

Sohail extended this to show how these dynamics intersect with expectations formed before migration. Sohail described deliberately lowering his expectations before arrival, anticipating barriers to professional recognition. Even with these adjusted expectations, he continued to perceive diminished status as something most clearly experienced through work. When asked to reflect on feeling out of place in Canada, he made a direct reference to his employment:

Look, I haven't felt like that directly, but most probably this is felt in your job.

Sohail's claim reinforces that occupational status is central to how belonging is experienced, even where expectations are adjusted in advance. For skilled racialized newcomers, symbolic capital loss is more than an economic barrier. It is a disruption of identity that reshapes how belonging itself is interpreted and experienced. Participants consistently compare their recognition in Pakistan with its absence in Canada, framing settlement as an ongoing negotiation of self‐worth and legitimacy. Belonging is therefore conditional on forms of recognition that are difficult to access, or unavailable altogether. This loss is not experienced solely as external exclusion, but as something that must be accommodated. As the following section shows, participants describe this process through the language of *lachak*, framing adjustment as an expected and necessary part of settlement.

### 5.2 “If You Don't Have That Lachak”: Flexibility as a Requirement of Settlement

If the previous section showed how symbolic capital loss disrupts professional identity, belonging, and a general sense of self, narratives in this section show how participants interpret these disruptions through *lachak*. From a Bourdieusian perspective, *lachak* can be understood as the lived expression of symbolic violence, through which misrecognition is accommodated and normalized during settlement. Across interviews, *lachak*, or flexibility, was repeatedly invoked as a necessary condition of settlement in Canada. What appears as an individual coping strategy both reflects and obscures broader institutional conditions and the sense of nonbelonging that emerges from these conditions.

Much of this process begins with newcomers confronting barriers that leave them with little choice but to adjust. Hussain, for example, explained that the loss of symbolic capital positioned him in an ongoing process of requalification:

All of my educational credentials, up until now, are Pakistani. In terms of my foreign qualifications—oh, I have an ACCA, but I didn't have to pass an exam for that. And now my plans are to become a CPA here. Right now, I'm in the process of going for that right now.

For Hussain, “the process” is not simply a bureaucratic step but a persistent contradiction. His narrative shows how recognition is contingent on forms of capital validated within the Canadian context. While meaningful to him, his Pakistani qualifications lack the same legitimacy in Canada. As a result, settlement becomes tied to repeated efforts to re‐establish credibility through Canadian institutions. He later framed this transformation in explicitly national terms:

I'm by birth Pakistani, who is in the transformation to be a Canadian […].

Hussain's emphasis on “transformation” underscores how belonging is experienced as something that must be earned through continual adjustment. Rather than being recognized for the expertise he had already accumulated, Hussain understood settlement as requiring him to re‐prove his worth through Canadian credentials and experiences. He went on to mention the need for “*lachak*” in this transformation. He noted that, irrespective of the privileges one may have enjoyed in Pakistan, “when you come here, you'll have to work on $14 an hour. That is going to be such a large *shock* for you that you won't be able to get through it if you don't have that *lachak* (participant's emphasis).” More than just economic dislocation, this “shock” reflects what Silva (2016) terms a “fragmented habitus,” where expectations and identities formed in Pakistan collide with their devaluation in Canada. The tension of such a fragmentation is not resolved with eventual settlement, it is managed, as immigrants accommodate these contradictions in order to move forward.

As with Hussain, these adjustments were framed explicitly through the need for flexibility. Rukhsana, for instance, described how the settlement environment itself inculcates this orientation:

The most important thing is that you need to set your mindset that there is a tremendous amount of struggle here, in the start. And even if you don't have that, the system over here will teach that to you itself [chuckles]. Secondly, like, over here, in terms of odd jobs…I've met a lot of people here that were highly educated in Pakistan. But when they came here with their families, they spent their entire lives working odd jobs just to make sure their families were set up here. This is a major drawback. That people just settle in order to set themselves up here, they just have to accept whatever job they can get. The odd jobs here are so tough, and then people just spent their entire lives in those jobs. Then they can't really escape that loop, even if they try.

Rukhsana's account highlights how flexibility is not simply a personal disposition, but something produced through institutional and labor market conditions. Her remark that “the system over here will teach that to you itself” suggests that symbolic violence is embedded within the settlement process itself, where adjustment is understood as a taken‐for‐granted feature of settlement, legitimizing misrecognition. For highly educated and skilled immigrants, the need to accept “whatever job they can get” reflects how symbolic capital loss is normalized as a routine feature of settlement. Rather than temporary setbacks, these outcomes are interpreted as an enduring feature of settlement in Canada that must be accommodated.

Dilawar similarly framed settlement through the language of flexibility through adaptation, drawing on a metaphor of rebirth to describe his experience:

We have learned about life twice. Graduate twice, grow up twice, and start over from nothing twice. In a way, be born twice. And what does that teach you? It teaches you that you have to face whatever happens. And for that to happen, you have to be adaptable. If you want to cope, you have to be flexible. Because if you don't do that, you can't survive here.

Dilawar's description of “being born twice” captures the depth of transformation that symbolic capital loss can entail, where settlement is experienced as a reconfiguration of identity, career trajectory, and expectations of recognition. Aamir's account illustrates the physical and emotional strain of this transformative process. Having worked in “management” in Pakistan, he described his early settlement period in Canada, marked by multiple low‐status, physically strenuous jobs, as a form of rebirth:

Listen, what I think is that Canada has given me another birth (*Canada ne mujhay reborn kiya hai dobara*). The challenges in Pakistan…in the beginning of my life…the challenges that I didn't face there, those I saw after I came here […]

Earlier in the conversation, Aamir described working through “fourteen odd jobs. Fourteen hard jobs,” including the kind of physically demanding manual labor that he had never performed in Pakistan. His description reveals how flexibility often involves enduring forms of work that stand in stark contrast to immigrants’ prior professional identities. Although he framed this experience positively through the language of rebirth, the repetition of precarious and physically demanding jobs underscores the consequences of symbolic capital loss, as material and physical adaptation becomes an embodied cost of adjustment that reshapes both lived conditions and sense of self. That Aamir frames this transformation as positive further illustrates how these conditions are normalized and absorbed into settlement, obscuring the nonbelonging they produce.

Finally, Faizan articulated the contradiction between Canada's immigration selection criteria, and the realities newcomers encounter after arrival:

*Bhai* (brother; ‘buddy’ as in this context), you can at least tell us. You're giving us points for it there, and here, there's a different world that you see. I mean, where is the consistency? Does the degree change when you come to Canada? Does the expertise? And when this happens, you have no option but to adjust. And now that we're in Canada, we'll adjust. If you want to live here, you'll have to adjust. Because no matter what happens, we're the ones that have to adjust. *Chalo* (irrespective of; ok), no matter what they said, now that we're here, we'll adjust as well.

Faizan's account reflects the central contradiction of the settlement structure. The same credentials that facilitated his entry into Canada lost their value upon arrival. Despite recognizing this inconsistency, he ultimately has no choice but to frame adjustment as inevitable. His repeated emphasis that “we're the ones that have to adjust” reveals how flexibility becomes an obligation, obscuring the structural contradictions that produce it.

Across these accounts, *lachak* emerges as both an interpretive framework and a mechanism through which structural conditions are reproduced. While participants describe flexibility as necessary for navigating the loss of professional recognition, it also reframes misrecognition as an individual responsibility to manage. In doing so, diminished recognition is incorporated into everyday understandings of settlement, allowing nonbelonging to persist while remaining difficult to identify as such.

### 5.3 Gendered Lachak: Uneven Adjustment and the Reorganization of Belonging

If *lachak* captures how newcomers adjust to the loss of symbolic capital, narratives from the women with whom I spoke show that these adjustments are not experienced uniformly. While men more often described downward mobility within the labor market, women more frequently spoke to *lachak* through the simultaneous loss of professional identity and the need to adjust to new domestic and familial roles. These shifts were also shaped by class and socioeconomic position, as migration reorganized not only paid work but also household labor, access to domestic support, and everyday status. As women struggled to enter the workforce in Canada, they described a restructuring of social roles that did not necessarily translate into greater recognition or autonomy, instead reproducing existing hierarchies in new forms.

Kashaf's account was illustrative of how these dynamics unfold across both professional and linguistic domains in the family. With a Master's degree in teaching from Pakistan, and aspiring to become a lecturer, she described teaching as a “craze” and a central part of her identity. Yet in Canada, she struggled to pursue this path while also managing domestic responsibilities and navigating language barriers. Reflecting on her experience, she explained:

My daughter is Canadian, at least more Canadian than me. I can't do the one thing I love to do here. I can't play those teaching games with my daughter like I used to. Your language is a major factor. Over there (in Pakistan), that was our language. English might be my daughter's language, but it will never be mine.

Kashaf's narrative demonstrates that symbolic capital loss extends beyond formal employment into intimate and relational domains. In addition to her career trajectory, her reduced ability to mobilize her linguistic and professional skills also impacts her relationship with her daughter, a social and domestic role for which she takes more responsibility than her husband. While her daughter becomes more incorporated into Canadian society, Kashaf experiences her own position as increasingly marginal. *Lachak*, in this context, involves adapting to a reconfiguration of familial roles and generational dynamics, revealing how belonging in settlement is unevenly distributed even within the same household.

Like Kashaf, Yasmin and Aisha—both former teachers in Pakistan—described how migration required them to relinquish their professional identities and take on exclusively domestic roles for the first time. In doing so, they framed their adjustment through *lachak* while also linking it directly to their sense of belonging in Canada.

Yasmin explained:

Over here, I don't really work. You know how it is. It's like, *we* had to understand what it's like for women in Pakistan after coming to Canada (chuckles). So, there is that adjustment, but you manage. But the truth is, it does make me think about Canada in a different way.

While migration is often associated with expanded opportunities, Yasmin instead describes learning to inhabit a more restricted role. Her use of “we” signals a gendered collectivity, referring not to newcomers broadly but to women specifically, who are expected to absorb these shifts through domestic and relational labor in settlement. Her account also points to the socioeconomic dimension of this adjustment, as the loss of paid work changes not only her employment status but also her position within the household. The need to “adjust” is framed as manageable, yet it subtly reshapes how she perceives Canada itself. *Lachak* here normalizes diminished recognition as an expected condition of settlement, reshaping how belonging is experienced. Aisha similarly linked her adjustment to both domestic labor and a diminished sense of self:

Over there (in Pakistan), you know, we had everything. We had house‐help with domestic chores. Now I do most of that. Since we decided that my husband will look for work first, I'm managing most of that. In Karachi, you know, you must've seen it, it's so easy to get that help. But here, I've become a *maasi* (a paid domestic worker) from being a teacher. So because of that, when you feel bad, you associate that with here as well.

Aisha's comparison between being a teacher and becoming a “maasi” highlights the nuance of this transformation. Her narrative shows how *lachak* requires women to absorb both professional and social demotion in ways that directly inform their nonbelonging. This demotion is also tied to class and socioeconomic status. In Karachi, access to paid domestic help allowed some middle‐class women like Aisha to sustain professional identities while delegating household labor. In Canada, the loss of that support reorganized household labor along gendered and socioeconomic lines, requiring Aisha to absorb domestic work in ways that intensified the loss of recognition. The affective dimension of this shift shows how feelings of frustration and loss are also attached to place itself, shaping how Canada is experienced in relation to Pakistan, requiring the acceptance of both symbolic and emotional loss.

While Aisha's account emphasizes loss, others described *lachak* as marked by contradiction, where gains in some domains coexist with losses in others. For example, Mehwish described gaining certain forms of personal autonomy in Canada while simultaneously experiencing a profound loss of professional recognition:

[…] You tell me, how can you feel like you're fully Canadian when you're coming from there from such a privileged position and here you feel like you have no worth, because you don't recognize yourself in this position.

Mehwish's reflection highlights a key contradiction in how *lachak* is experienced. While she described increased personal freedom that she did not experience in Pakistan, this did not alter her sense of nonbelonging. Instead, it was overshadowed by the erosion of the professional identity and socioeconomic position that had previously anchored her sense of self. *Lachak* does not produce a straightforward process of adjustment, but rather a reconfiguration of what counts as meaningful recognition. Gains in one domain do not compensate for losses in another, revealing how belonging remains uneven and contingent, even where certain forms of inclusion or autonomy are gained.

While these adjustments unfold differently across individuals, their normalization was not only learned after arrival but often anticipated in advance. Jamila, a teacher in Karachi who migrated with her husband and children, described how expectations of downward mobility were communicated to her prior to migration:

Look, this is what you have to do; I think even they (her relatives with whom they're living right now) told us that this is just what everyone does. You have to go through it, so we were ready for that. We used to talk to them before coming, so we were prepared for it. Now, it's not going to be like, that you come here and right away you start teaching.

Jamila's account reveals that *lachak* is not simply reactive but anticipatory, shaped through transnational networks that normalize adjustment as an expected part of migration. When asked whether these experiences affected her sense of belonging, she reflected:

Honestly, we don't really have the time to think about that. I mean, I'm sure it does. In the back of your mind probably, but right now we have to think about surviving. And then once we're settled here then we'll see. Meaning, if you're asking me whether I'm Canadian, I know that I am. That is for sure. We're here. Pakistanis and Canadians. But I used to really like having a job, and being a teacher, but that's not probably likely for me here, that I'll get to do that. You know what it's like for women in the family. You have to adjust, and that obviously will affect you somewhat I guess. But whatever the case, we'll have to do it. What other option do we have.

Her response highlights a central tension across participants’ narratives. Belonging is affirmed at the level of formal identity yet constrained in practice by the limited recognition available to them. The urgency of economic survival displaces the question of belonging itself, rendering it secondary to the immediate demands of settlement. At the same time, her reference to “what it's like for women in the family” shows how these adjustments are shaped by gendered expectations, where the loss of professional identity is absorbed through a reorientation toward domestic and relational roles. For women, *lachak* reveals how the temporal structure of belonging is reorganized, pushing its realization into an indefinite future. Consequently, as belonging is delayed and becomes uncertain, the need for adjustment remains immediate and unavoidable.

Across these accounts, *lachak* emerges as a gendered condition through which immigrants come to inhabit unequal positions within Canadian society and within their own households. While flexibility is required of all newcomers, its terms are unevenly distributed across professional, domestic, relational, and socioeconomic domains. *Lachak* thus shows how diminished recognition is normalized through everyday adjustment, but in ways that are structured by gender, class, and socioeconomic position.

## 6 Discussion and Conclusion

This study shows how skilled Pakistani newcomers interpret the loss of professional recognition as an enduring feature of settlement rather than a temporary disruption. It extends existing research on credential devaluation and deskilling among racialized immigrants (Banerjee et al. 2024; Bauder 2003; Guo 2015) by showing how these conditions are incorporated into immigrants’ everyday understandings of settlement. Rather than treating adjustment as a temporary response to structural barriers, participants framed the need for *lachak*, or flexibility, as an expected and necessary feature of settlement. In doing so, conditions of misrecognition are not only endured but absorbed into how immigrants understand their place in Canada. This shifts attention from adaptation as a response to inequality, to a mechanism through which these conditions are sustained.

These findings contribute to scholarship on belonging and migration by demonstrating how nonbelonging persists alongside formal inclusion. Building on Yuval‐Davis’ (2006; 2011) concept of the politics of belonging through Korteweg and Yurdakul's (2024) formulation of nonbelonging, the analysis shows how membership in the national community is experienced as conditional and negotiated. Rather than operating solely through exclusion, nonbelonging is sustained through everyday practices of adjustment that enable immigrants to maintain their conditional and negotiated place within the national community.

Bringing this into conversation with Bourdieu's framework highlights how these processes are internalized. The devaluation of foreign credentials operates not only as institutional misrecognition but as a form of symbolic violence, as immigrants come to interpret adjustment as necessary (Bourdieu 1984; 1991). Structural inequities are thus reproduced through the normalization of responses to those constraints. This extends existing applications of Bourdieu in migration scholarship (Huot 2017; Ibrahim 2022) by showing how symbolic violence operates through everyday practices of settlement.

The findings also demonstrate that these processes are unevenly structured by gender, class, and socioeconomic position. For the women in this study, *lachak* was experienced through the reorganization of domestic roles, household labor, and everyday responsibilities. While men more explicitly framed their experiences in terms of downward mobility within professional fields, women's accounts more readily connected these dynamics across both professional and social domains. In addition, the reorganization of domestic and relational roles emerged primarily in women's narratives, highlighting how flexibility extended beyond the labor market in gendered ways. This extends existing research on gendered labor market inequality (Amoako et al. 2024) by showing how these dynamics reshape broader forms of recognition and belonging.

This study contributes to migration studies and Canadian sociology by showing how adjustment itself sustains conditions of nonbelonging. By foregrounding *lachak* as an analytic, it demonstrates how adjustment operates as both a necessity and a constraint, enabling settlement while reproducing and obscuring these dynamics. More broadly, these findings suggest that addressing inequities in immigrant settlement requires moving beyond a focus on labor market outcomes alone. As long as diminished recognition is treated as an expected cost of settlement, structural exclusion and inequities will continue to be reproduced in ways that remain difficult to identify and contest. This aligns with broader critiques of policy approaches that prioritize economic integration while overlooking lived experiences of recognition and belonging (Bloemraad 2015; Thomas 2023).

Despite these contributions, this study has several limitations that point to directions for future research. First, the analysis focuses on a single national‐origin group within the GTA, which, while analytically strategic, does not capture the full range of immigrant experiences across contexts. Comparative work across immigrant groups and regions would help assess the broader applicability of these findings. Second, this study centers immigrants’ perspectives and does not examine the role of institutional actors, such as employers or credentialing bodies, whose practices shape these conditions. Future research integrating these perspectives would further illuminate how misrecognition is produced and sustained institutionally. Finally, while this study highlights how nonbelonging is normalized, future work could examine how these processes are resisted, contested, or reinterpreted over time, particularly across stages of settlement or generations.

## Funding

This research was supported by the Social Sciences and Humanities Research Council of Canada (SSHRC) Doctoral Fellowship [SSHRC Ref.: 752‐2018‐2477].

## Ethics Statement

This research was approved by the University of Toronto's Ethics Board.

## Conflicts of Interest

None of the authors have a conflict of interest to disclose.

## Endnotes

1
*Lachak* () is an Urdu term commonly translated as “flexibility,” but it includes connotations of elasticity, pliability, and the capacity to bend or adjust under pressure. In everyday usage, it can also refer to the ability to accommodate changing circumstances or endure hardship by adapting one's expectations and behavior. In this article, I use *lachak* as participants deploy it, as a moral and practical orientation through which diminished recognition is accepted and normalized as part of the settlement process.

2 The Urdu phrases “*aap ko toh pata hai*” and “*aap toh jaantay hain*” both loosely translate to “you already know,” “you know what it's like,” or “you understand,” and are commonly used to signal assumed shared understanding, particularly in contexts where speaker and listener are presumed to share similar social or cultural experiences.
